# Nematocidal Potential of Phenolic Acids: A Phytochemical Seed-Coating Approach to Soybean Cyst Nematode Management

**DOI:** 10.3390/plants13020319

**Published:** 2024-01-21

**Authors:** Ping Yates, Juddy Janiol, Changbao Li, Bao-Hua Song

**Affiliations:** 1 Department of Biological Sciences, University of North Carolina at Charlotte, 9201 University City Blvd, Charlotte, NC 28223, USA; pyates4@uncc.edu (P.Y.);; 2 Syngenta Crop Protection LLC, 9 Davis Drive, Durham, NC 27709, USA

**Keywords:** *Heterodera glycines*, botanical nematicide, biopesticide, organic farming, environmental sustainability

## Abstract

Soybeans, one of the most valuable crops worldwide, are annually decimated by the soybean cyst nematode (SCN), *Heterodera glycines*, resulting in massive losses in soybean yields and economic revenue. Conventional agricultural pesticides are generally effective in the short term; however, they pose growing threats to human and environmental health; therefore, alternative SCN management strategies are urgently needed. Preliminary findings show that phenolic acids are significantly induced during SCN infection and exhibit effective nematocidal activities in vitro. However, it is unclear whether these effects occur in planta or elicit any negative effects on plant growth traits. Here, we employed a phytochemical-based seed coating application on soybean seeds using phenolic acid derivatives (4HBD; 2,3DHBA) at variable concentrations and examined SCN inhibition against two SCN types. Moreover, we also examined plant growth traits under non-infected or SCN infected conditions. Notably, 2,3DHBA significantly inhibited SCN abundance in Race 2-infected plants with increasingly higher chemical doses. Interestingly, neither compound negatively affected soybean growth traits in control or SCN-infected plants. Our findings suggest that a phytochemical-based approach could offer an effective, more environmentally friendly solution to facilitate current SCN management strategies and fast-track the development of biopesticides to sustainably manage devastating pests such as SCN.

## 1. Introduction

Soybean (*Glycine max* (L.) Merrill), one of the most economically valuable agricultural crops worldwide, contributes to the majority of plant-derived proteins, fats, and oil products. However, soybean crops are globally decimated by the soybean cyst nematode (SCN; *Heterodera glycines* Ichinohe), a parasitic pest resulting in massive annual losses in soybean yields and economic revenue (~$1.5 billion in the U.S. alone) [1,2,3]. Plant parasitic nematodes (PPNs), such as SCN, can inflict significant harm on plants by ciphering off nutrients, depleting water availability, and consequently, making them more susceptible to further pathogenic infections or pest-induced damage [4]. While conventional agricultural practices (such as synthetic pesticides) are generally effective in the short term, these approaches also have a long history of being associated with hazardous effects on human health and the environment. As a result, public opinions in recent years have favored alternative pest management strategies, and thus have prompted the growing demand for more sustainable, and safer, solutions [5,6,7]. In conjunction with traditional farming practices, SCN management relies heavily on the development and breeding of improved SCN-resistant soybean varieties to combat expanding SCN populations [2,3]. However, the effectiveness of resistant cultivars which are currently employed is decreasing due to SCN evolution while efforts to develop more effective SCN-resistant cultivars requires a considerable amount of time, rigorous regulatory approval, and has sizable financial costs [2,3].

In the last couple of decades, seed coating technology has gained increasing recognition as a lower-input, efficient, and more cost-effective approach to delivering agronomic treatments to aid pest control and improve crop productivity [8]. Seed treatment applications, both chemically and biologically driven, are emerging as promising management tools for reinforcing plant defenses against multiple biotic threats, such as nematode infection, while enhancing overall plant health [9,10,11,12,13,14,15,16,17]. Currently, most progress toward applied seed treatments has been made by developing chemical nematicides that can be exogenously applied to crop seeds through various methods. Notably, a handful of these chemical nematicides are already commercially available through major companies such as Bayer, Syngenta, and BASF. However, the majority of these products are comprised of synthetic pesticides such as fluopyram, a widespread fungicide more recently utilized as a nematicide [5,18,19]. Even though fluopyram is highly effective, it also is associated with negative effects including disruptive impacts on soil microbial communities, environmental persistence and potential accumulation, the creation of toxic byproducts, and possiblelinks with liver cancer in a mammalian model [18]. Despite the success of synthetic nematicides and our dependency on them in current agricultural systems, it is urgent that we take drastic steps to shift our research efforts toward alternative nematicides with substantially minimized impacts on environmental and human health.

In the pursuit of developing such novel nematode control strategies, much research has focused on studying plants’ immune responses and defense mechanisms, particularly those involving secondary metabolites [4]. The production of these defensive phytocompounds can be categorized as constitutive (phytoanticipins) or induced (phytoalexins) in which the presence of exogenous organisms or compounds can signal the induction of plant immune responses and defensive measures [4,20,21]. As global trends in pesticide use gradually shift away from conventional approaches, biopesticides and phytochemical pesticides are emerging as promising and more-sustainable alternatives [5,6,7,22,23,24,25,26,27] in conjunction with diverse application methods such as seed coating techniques [8,9,28,29,30,31]. Notably, the application of phytochemical or botanical nematicides [6,7,24,26,28,32,33,34,35,36,37,38,39,40,41,42,43,44] has garnered increasing research interest due to their attractive qualities over conventional pesticides, such as lower toxicity, higher target specificity, lower environmental persistence, and biodegradability [6].

Among many major classes of allelochemicals, known as plant defense compounds (terpenoids, saponins, alkaloids, glucosinolates), phenolic compounds, in particular, have long been associated with plant defense tactics, such as nematode resistance, [4,34,45,46,47] and have garnered further research interest for their extended potential in plant and human health [48,49,50,51,52]. In general, phenolics include more complex polyphenol and oligophenol compounds and simpler monophenol compounds [53]. Simple phenolic acids, such as benzoic acids and its derivatives, are among the most widely and abundantly distributed compounds in plants and surrounding rhizosphere through root exudation [21,53]. Notably, previous findings have revealed that multiple simple phenolic acids accumulate in SCN-resistant soybean genotypes during the onset of SCN infection and demonstrate in vitro nematocidal activity (Song Lab). However, it has yet to be determined whether these compounds exert inhibitory effects in planta and whether they may trigger any unintended negative effects on plant growth and development, particularly when exposed to higher phytochemical doses. In conjunction with the lack of data exploring the potential of botanical nematicides in planta, there is also a lack of public data on the efficacy of seed coating treatments despite the widespread use of seed treatments [54] and a growing global market (multiple billions annually) [55]. To address these questions, we employed a phytochemical-based seed coating application to soybean seeds using phenolic acid derivatives, 4-hydroxybenzaldehyde (4HBD) and 2,3-dihydroxybenzoic acid (2,3DHBA), at variable concentrations and assessed the resulting effects under control and SCN-infected conditions (Race 3, HG type 0; Race 2, HG type 1.2.5.7). Both 4HBD and 2,3DHBA naturally occur in a broad array of plants and have applications as flavoring agents/adjuvants, fragrance components [56], and as a matrix compound for biochemical analyses [57]. Not only do these organic compounds show potential as safer agrichemicals in contrast to conventional pesticides, but they also confer therapeutic effects associated with a number of human disorders and diseases [56,57]. In this study, we are among the first to demonstrate the use of phytochemical treatment in a soybean-SCN pathosystem as an alternative method of nematode control using a simplified seed coating technique.

## 2. Results

### 2.1. Phytocompound Effects on Soybean Growth Traits

**Figure 1:**
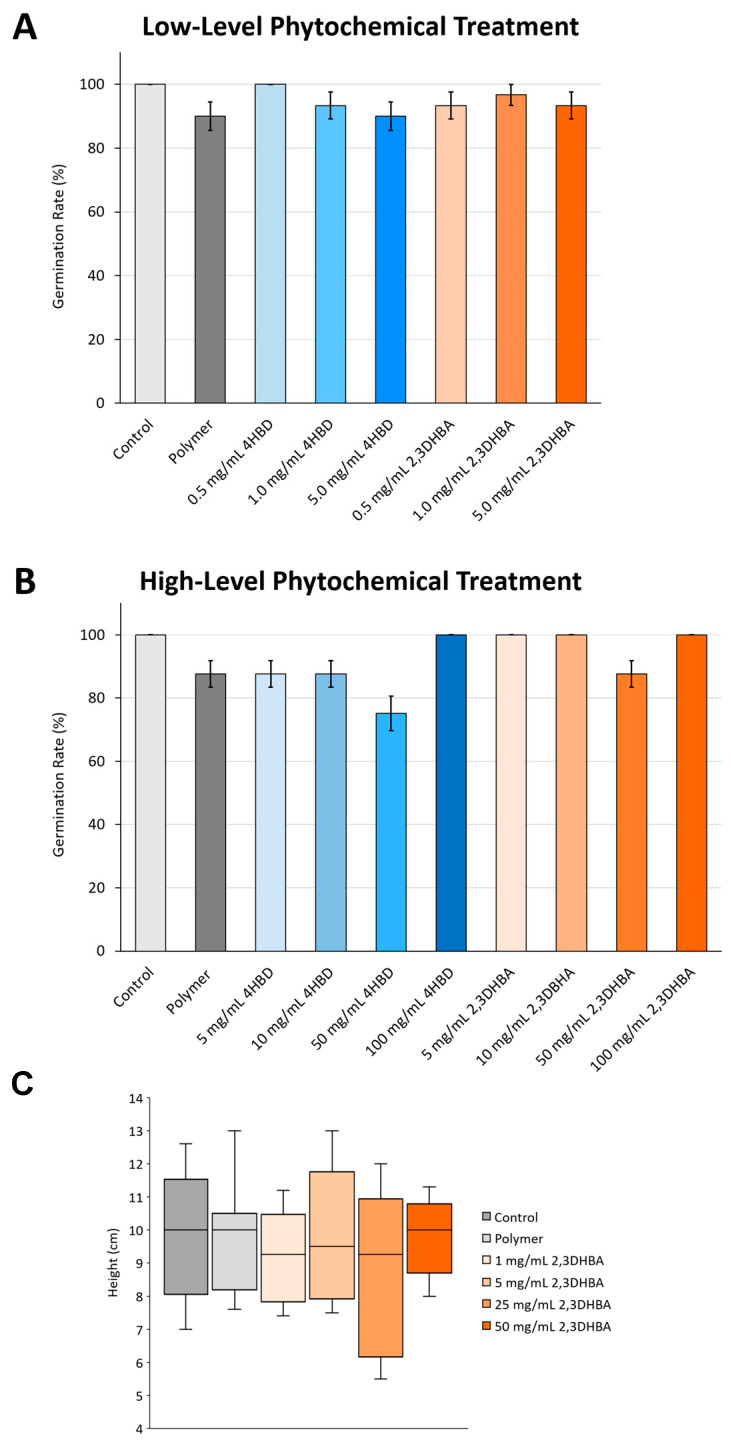
Quantified effects of studied phytochemicals (4HBD, blue; 2,3DHBA, orange) on soybean seed germination rate (% mean ± sd), as well as 2,3DHBA on early growth (2-week-old seedlings; height in cm). Light grey: untreated (control) seeds; Dark grey: polymer-treated seeds; Blue: 4HBD-treated seeds; Orange: 2,3DHBA-treated seeds. Neither low-level (**A**) nor high-level phytochemical treatments (**B**) resulted in any significant reduction in soybean germination success compared to untreated controls (one-way ANOVA; *p* > 0.05). Neither 2,3DHBA treatment or polymer treatment resulted in significant reductions in soybean plant growth compared to the control ((**C**), mean ± sd; n = 8–10 per treatment; one-way ANOVA; *p* > 0.05).

Soybean seeds treated with a series of concentrations of 4HBD or 2,3DHBA, as well as those only coated with a polymer, did not result in any significant reduction in seed germination rates (% mean ± sd; one-way ANOVA; *p* > 0.05) when compared to the control group (non-coated seeds). Here we examined a range of both low-level (0.5, 1.0, 5.0 mg/mL) (Figure 1A) and high-level (5.0, 10.0, 50.0, 100.0 mg/mL) phytochemical treatments for each compound (Figure 1B).

Next, we asked if phytochemicals affect plant early development. We evaluated plant height at 2 weeks old using seeds coated with different concentrations of 2,3DHBA (1.0, 5.0, 25.0, 50.0 mg/mL). The reason that we only used 2,3DHBA in this assessment is because it showed greater potential for SCN control than 4HBD (see Section 2.2). The results showed that there was no significant reduction in early plant growth between the pre-treatment of seeds with 2,3DHBA and the control group (mean ± sd; n = 8–10 per treatment; one-way ANOVA; *p* > 0.05; Figure 1C).

**Figure 2:**
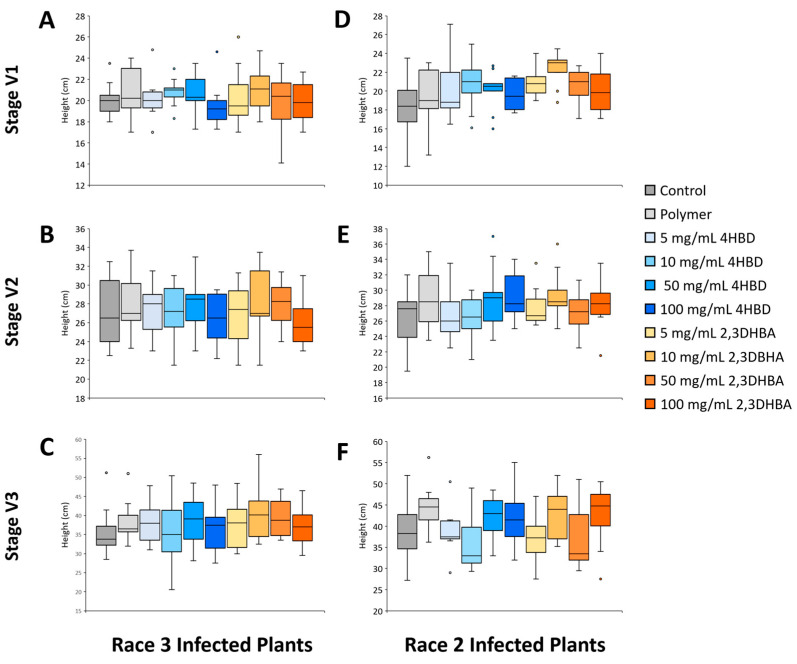
Phytochemical effects (4HBD or 2,3DHBA) on plant growth (height, mean ± sd) under nematode infection during early developmental stages (V1, V2, V3). (**A**–**C**): Race 3 treatment (n = 11–14 per treatment; *p* > 0.05). (**D**–**F**): Race 2 treatment (n = 8–12 per treatment; *p* > 0.05).

Third, we quantified plant growth (plant height) at early stages of development (V1–V3) under SCN infection (Race 2 and Race 3, respectively) using phytochemically treated (4HBD or 2,3DHBA) soybean seeds at varying concentrations (5.0, 25.0, 50.0, 100 mg/mL). In both Race 2 (n = 8–12 per treatment) and Race 3 (n = 11–14 per treatment) infected plants, neither 4HBD or 2,3DHBA treatments resulted in any significant decreases in plant height at any developmental stages compared to control groups (one-way ANOVA; *p* > 0.05; Figure 2).

### 2.2. Phytocompound Inhibitory Effects on SCN Abundance

**Figure 3:**
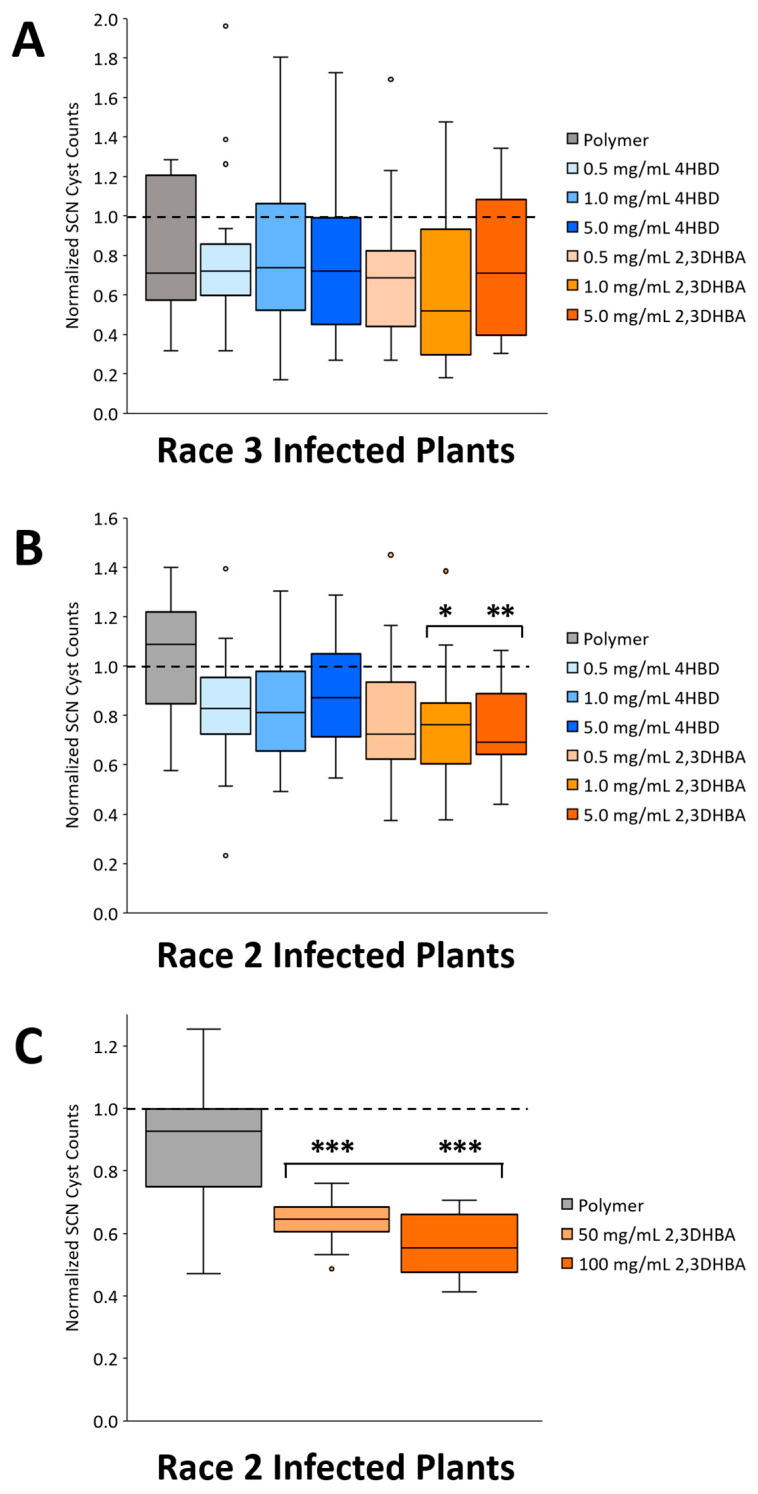
Phytochemical effects (4HBD or 2,3DHBA) on nematode control (nematode cyst counts at 30 dpi; mean ± sd) in SCN-infected (Race 3 or Race 2) soybean plants (~6-week-old). (**A**) SCN cyst counts were not significantly reduced under low concentrations of either 2,3DHBA or 4HBD in Race 3 infected plants (n = 13–19; Kruskal–Wallis test; *p* > 0.05). (**B**) SCN cyst counts were significantly reduced under low concentrations of 2,3DHBA (n = 14–18 per treatment; one-way ANOVA, *p* < 0.05; Dunnett’s test, *p* < 0.05) but not under low concentrations of 4HBD in Race 2 infected plants (one-way ANOVA; *p* > 0.05). (**C**) SCN cyst counts were significantly reduced under higher concentrations of 2,3DHBA in Race 2 infected plants (n = 6–10 per treatment; Welch’s ANOVA, *p* < 0.05; Dunnett’s T3 test, *p* < 0.05). Dashed horizontal line (1.0) signifies the control group average in which the values of the treatment groups have been normalized accordingly to convey increases or decreases in relation to the control. Asterisks (*) denote statistical significance: * *p* < 0.05; ** *p* < 0.01; *** *p* < 0.001.

Nematode cyst abundance was examined in SCN-infected soybean plants 30 days post inoculation (dpi) to assess nematode inhibitory potential of phytochemically coated seeds (4HBD or 2,3DHBA). Under lower-concentration 4HBD treatments, nematode cyst counts were not significantly reduced (one-way ANOVA or Kruskal–Wallis test; *p* > 0.05) between the control or treatment groups in either Race 3 (n = 13–19 per treatment; Figure 3A) or Race 2 infected plants (n = 14–18 per treatment; Figure 3B).

In contrast, under lower-concentration 2,3DHBA treatments (1.0, 5.0 mg/mL), nematode cyst counts were significantly reduced in Race 2 infected plants (n = 14–18 per treatment; one-way ANOVA, *p* < 0.05; Dunnett’s test, *p* < 0.05; Figure 3B) but not in Race 3 infected plants (n = 13–19 per treatment; Kruskal–Wallis test; *p* > 0.05; Figure 3A). To test the hypothesis that a higher concentration of phytochemicals would increase SCN control (e.g., further decrease cyst counts in the roots), a secondary SCN-inhibitory assay was pursued with only 2,3DHBA. The reason 4HBD was excluded in this assay was due to its lack of nematocidal potential in earlier tests (Figure 3A,B), whereas 2,3DHBA showed greater nematocidal potential under lower concentrations. Furthermore, under a higher-concentration of 2,3DHBA treatments (50.0, 100.0 mg/mL), tested only in Race 2 infected plants, nematode cyst counts were significantly reduced (n = 6–10 per treatment; Welch’s ANOVA, *p* < 0.05; Dunnett’s T3 test, *p* < 0.05; Figure 3C) compared to the control group.

## 3. Discussion

In this study, we demonstrate that seed-coating using simple phenolic compounds can serve as an effective means of nematode control against select SCN races, without significant detriments to plant early growth. More specifically, our results indicate that 2,3DHBA significantly reduces SCN abundance (indicated by cyst counts) in Race 2-infected soybean plants in a dose-dependent manner. As such, the nematocidal effects elicited by 2,3DHBA illustrate its potential as a candidate phytochemical tool to assist SCN management in soybean. Our investigation contributes to the limited, but growing, number of studies that have examined the nematocidal potential of phytochemicals in planta in conjunction with their effects on plant growth parameters. Moreover, to the best of our knowledge, this study is the first to investigate the specific effects of phenolic acids 4HBD and 2,3DHBA in a soybean–SCN plant pathosystem. Although there is a lack of reports regarding 4HBD or 2,3DHBA specifically, a handful of studies [34,39,45,49,58,59,60] have tested comparable phenolic acids for their nematocidal potential. Notably, relatively low doses of phenolic acids 3,4DHBA [39], 3,5DHBA [58] and 2,6DHBA [45] reportedly elicit moderate to high nematocidal activity (>90%) against root knot nematodes *Meloidogyne incognita* and/or inhibit egg hatching under in vitro conditions. Likewise, various other phenolic acids, such as phenols and nitrophenols, also demonstrate strong in vitro nematocidal activity or toxicity against *M. incognita,* [34,36,39,45,58] *H. glycines,* [59] and *Heterorhabditis bacteriophora* [43]. Furthermore, compound dosage and nematode exposure time have been tested to evaluate nematicide efficacy through in vitro assays where, similar to our findings, phytochemical nematocidal effects appear to be concentration-dependent [34,42,49,58,59]. Interestingly though, the review of studied cases [4] also suggests that specific compounds may be strongly correlated with resistance to nematode infection, even in low concentrations, whereas entire classes of metabolites, regardless of their total concentration, may be uncorrelated to nematode control, suggesting phytochemical-specific effects. However, despite the growing support for the role of simpler phenolic compounds as nematocidal agents, the majority of these reports generally rely on in vitro laboratory trials and, to a lesser degree, in planta greenhouse trials, whereas more definitive evidence is extremely scarce (mutagenesis-function studies, field trials) [4]. Overall, more work is needed to evaluate the potential of phytochemicals under in vitro and in planta conditions and their chemical behavior and practicality under field testing conditions for the application of phytochemicals in SCN management.

Recently, the use of metabolic markers has emerged as a promising tool to identify novel biomarkers [4]. Notably, the application of targeted metabolomics has been a suggested method to screen for biomarkers associated with nematode resistance in breeding cultivars to potentially replace conventional nematode resistance assays [4]. Although less commonly employed, an untargeted metabolomics approach can be useful for identifying putative nematocidal compounds that characterize different plant conditions (resistant vs. susceptible, constitutive vs. induced). After which, the role of such compounds in plant–nematode interactions may be followed up with in vitro or in planta testing and validated with knock-out mutants or RNAi approaches [4,47]. Although very limited, such studies have yielded a mixed array of results concerning differences in phenolic compound accumulation among nematode-susceptible and resistant plant cultivars under variable infected or uninfected states. However, in most of these cases, in vitro and in planta test-based evidence is generally lacking [4]. Furthermore, work aimed at understanding the underlying roles and interactions of phytochemicals in a biological-ecological context has employed multiple omics approaches including transcriptomics in conjunction with metabolomic profiling techniques (i.e., GC-MS, HPLC-MS, NMR) [49,61] to characterize differential phytochemical metabolism and corresponding phytochemical activities in soil environments [62,63]. Overall, reliable screening of putative biomarkers will depend on a multifaceted approach combining metabolic profiles and better predictive statistical tools [4,61].

In addition to exploring the nematocidal potential of phenolic compounds, other studies have characterized their effects on plant growth and development, with reported effects ranging from negative to negligible to positive [42,46,64,65,66,67,68,69,70,71,72]. Such contrasting outcomes are likely attributed to variable factors (dose-dependency, plant species, compound, growth medium) and therefore make comparisons difficult. For instance, the efficacy of phytochemicals applied in direct contact assays at lower doses may not be very comparable to phytochemical applications in greenhouse or field soil conditions that utilize much higher doses across a diffuse environment [6]. In particular, phenolic acids have been associated with phytotoxic effects on plant physiology, although the molecular targets of such phenolic acids still remain poorly understood [53]. Collectively, these reports suggest that the underlying cause of these phytotoxic effects stems from the interaction between phenolic acids and biological membranes. Consequently, the disruption of biological membranes can diminish cell membrane potential and transport, resulting in a number of negative secondary effects on plant functions (photosynthesis, respiration, stomatal regulation, hormone regulation), and ultimately lead to deficits in plant growth and development [53]. As reflected in the literature, the nature of phenolic compounds is multifaceted and complex, as evidenced by the mixed array of plant-benefiting and plant-damaging effects and, therefore, warrants further investigation to elucidate the mechanistic actions of both direct and indirect functional roles of phenolic compounds. Regarding our present study, we did not observe any significant increases or reductions in soybean growth and development pertaining to germination rates or plant height, although we did notice some generally increasing trends in plant height. It is possible that statistically significant increases in plant growth traits could be discerned with larger scale experiments using expanded sample sizes. Furthermore, the investigation of additional seed and plant growth parameters (germination time, shoot length/biomass, leaf abundance/development, stem diameter, photosynthetic efficiency/pigments) may provide clearer evidence of any beneficial impacts induced by 4HBD or 2,3DHBA.

Generally, plant secondary metabolites benefit overall plant growth and physiological health, either directly through plant stress tolerance/defense/developmental processes or indirectly by signaling the colonization of plant-growth-promoting microbes that foster synergistic plant community interactions, thereby benefiting the plant [63,73]. When considering the dual effects of phenolic acids on parasitic nematodes and plant health, research must also explore their extended impact on soil environments that harbor diverse microbial communities (bacteria, fungi, protists) in the rhizosphere. Phenolic compounds, which are considered to be the most ubiquitous plant allelochemicals, play an essential role in modulating the complex interactions within the rhizosphere [46]. The dynamic nature of soil environments (water content, chemical bonding, organic matter composition, pH) largely governs the fate of phenolic acids (unbound, irreversibly bound, or reversibly bound) and, therefore, influences their subsequent bioavailability, biodegradability, accumulation, and persistence [46,74]. As a result, attempts to elucidate the ecological (allelopathic, phytotoxic, anti-microbial) implications and direct/indirect roles of phenolic acids in the rhizosphere can be very challenging. Notably, various phenolic acids which are secreted as root exudates [59,74] can be readily metabolized by various soil microorganisms (bacteria, fungi) and, consequently, their presence can alter the diversity and composition of soil microbial communities resulting in the establishment of pathogenic or mutualistic interactions [29,75,76,77,78]. In soybeans for instance, various phenolic acids, such as benzoic acids, are exudated into the rhizosphere and can act as chemoattractants that facilitate quorum sensing activities in beneficial microbes, such as *Rhizobium* [70], which subsequently help protect against nematode infection (*M. incognita*) [29]. Although these findings suggest phenolic acids may positively impact soybean–SCN interactions, there is very little evidence surrounding their physical/chemical interactions under highly variable field conditions to assess their agricultural implications. As such, the potential application of such phytochemicals in dynamic soil environments [6] warrants further examination of their effects on soil communities and any tradeoffs regarding lower environmental persistence and efficacy as nematocidal agents.

Currently, effective nematode control presents a massive ongoing challenge as it becomes clear that progressive solutions necessitate an integrated management approach that encompasses a strategic combination of farming practices, resistant crop varieties, chemical applications, and biocontrol methods [2,3,4]. As research continues to investigate the pairing of phytochemical-based nematicides with seed application methods, there are a number of challenges and constraints that can slow progress, including compound identification, purification, product standardization, field trials, and regulatory barriers [6]. In addition to challenges in developing candidate phytochemical nematicides, there are still ongoing debates and knowledge gaps regarding the polarizing effects and multi-functional roles of phenolic acids, which appear largely context dependent. In part, this disparity likely stems from the difficulty in obtaining causative evidence that specific phytochemicals are directly linked to nematode resistance [4]. As such, it is necessary that research in this newly evolving field continues to investigate basic phytochemical effects under simple and more complex scenarios in order to finetune their application as future biomarkers for resistant breeding lines and exogenous application in nematode management practices.

Currently, conventionally used synthetic pesticides dominate the agricultural landscape despite the rapidly growing demand for organically produced crops. This is partly due to the inability of organic agricultural practices to commercially compete with more cost-effective, higher-producing, synthetic agricultural practices [25]. Therefore, it is necessary to identify novel strategies to boost the productivity and economic viability of organically cultivated crops. Amongst these efforts, exploring novel phytochemical, or botanical-based pesticides has the potential to contribute to the broad-spectrum protection of crops while bearing lower environmental impacts. Furthermore, integrated pest management approaches that employ strategic combinations of biopesticides, including biochemical and microbial-based pesticides, show much promise as alternative means of pest control [6,24] and, consequently, will extend the production capabilities of organically grown crops. In our present study, we showcase only a glimpse of the potential exhibited by one class of phytochemicals to enhance plant defense against nematode pests. The promising results for phytochemicals such as 2,3DHBA bode well for future investigations of diverse phytochemicals and their potential applications in sustainable agricultural practices [73].

## 4. Materials and Methods

### 4.1. Seed Treatments and Growth Conditions

Soybean (Williams 82 cultivar) seeds were coated with variable concentrations of either 2,3 dihydroxybenzoic acid (2,3DHBA) or 4-hydroxybenzaldehyde (4HBD) and compared alongside controls (uncoated seeds and polymer-coated seeds). Polymer (Germain’s Seed Technology, Norfolk, UK) mixtures for seed coating were prepared based on manufacturer’s recommendations according to seed weight (100 mg polymer/1 kg seeds). The phytochemicals used in this study were purchased as dry reagents from commercial vendors (4HBD from Thermo Fisher Scientific (Waltham, MA, USA) and 2,3DHBA from Sigma (St. Louis, MO, USA)) and prepared at various concentrations by dissolving each compound in deionized water, followed by mixing with an appropriate amount of polymer (1 part polymer; 2 parts water/compound solution) through rigorous vortexing in 50 mL plastic Falcon tubes. Control included non-coated seeds and polymer-coated seeds. All coated seeds were air-dried at room temperature overnight prior to planting.

For experiments involving early plant growth traits (germination, height), seeds were planted in a mixture of sterilized soil/sand (1 part sand; 2 parts soil) and maintained under growth chamber conditions (27 °C; 16:8 h light/dark cycle) for ~2 weeks.

For experiments evaluating SCN inhibition (indicated by the number of SCN female cysts), seeds were planted in plastic cones containing sterilized sand and maintained under growth chamber conditions (27 °C; 16:8 h light/dark cycle).

For longer-term experiments evaluating plant growth traits (height during V1–V3 stages) under SCN infection, seeds were planted in plastic cones containing a mixture of sterilized soil/sand (1 part sand; 2 parts soil) and maintained under growth chamber conditions (27 °C; 16:8 h light/dark cycle).

### 4.2. Nematode Extraction and Inoculation

For all SCN experiments, seedlings were inoculated with freshly extracted SCN eggs (either Race 2 or Race 3) 10 days post-planting. SCN eggs were extracted from laboratory SCN stock plants using standard procedures. The roots of individual plants were inoculated with ~2000 SCN eggs.

### 4.3. Data Collection

For early plant growth traits, seed germination rates and seedling growth (plant height, ~2 weeks post-planting) were recorded. For SCN inhibition experiments, nematode cysts for each SCN type were collected 30 days post inoculation (dpi) (~6-week-old plants), following which cyst counts were recorded. For plants grown under SCN infection, plant height was recorded during early soybean developmental stages (V1, V2, V3) as indicated by the emergence of key morphological features.

### 4.4. Data Analysis

For each experimental group, dataset averages and standard deviations (mean ± sd) were computed for all recorded parameters (germination rate, nematode cyst count, plant height). Datasets containing extreme values due to abnormal plant development/growth conditions were omitted from further analysis. In our experimental approach, we aim to investigate whether each phytochemical-treated group significantly differed from control groups, but we did not aim to assess whether compound effects differed between each other at variable concentrations. Therefore, under these analysis conditions, we define phytochemical treatment as the single factor being assessed among all experimental groups. Depending on which parametric testing criteria were met through diagnostic testing (normality, equality of variances), we proceeded to analyze data with a one-way ANOVA or Welch’s ANOVA (unequal variances) to detect any significant difference among experimental groups. Alternatively, if datasets did not adhere to a normal distribution, datasets were either transformed or analyzed with the Kruskal–Wallis test (non-parametric). If either ANOVAs or Kruskal–Wallis tests yielded a significant result, we proceeded testing with either Dunnett’s or Dunnett’s T3 post hoc test to identify significant differences between controls and specific treatment groups. All statistical analyses were performed using IBM SPSS software (v14) and figures were constructed using Microsoft Excel and PowerPoint (Office 2016). An alpha level of (*p* < 0.05) was used to denote statistical significance.

## Data Availability

Data are contained within the article.
